# Two-step paretial least square regression classifiers in brain-state decoding using functional magnetic resonance imaging

**DOI:** 10.1371/journal.pone.0214937

**Published:** 2019-04-10

**Authors:** Zhiying Long, Yubao Wang, Xuanping Liu, Li Yao

**Affiliations:** 1 State Key Laboratory of Cognitive Neuroscience and Learning, Beijing Normal University, Beijing, China; 2 School of Information Science and Technology, Beijing Normal University, Beijing, China; University of Pennsylvania Perelman School of Medicine, UNITED STATES

## Abstract

Multivariate analysis methods have been widely applied to decode brain states from functional magnetic resonance imaging (fMRI) data. Among various multivariate analysis methods, partial least squares regression (PLSR) is often used to select relevant features for decoding brain states. However, PLSR is seldom directly used as a classifier to decode brain states from fMRI data. It is unclear how PLSR classifiers perform in brain-state decoding using fMRI. In this study, we propose two types of two-step PLSR classifiers that use PLSR/sparse PLSR (SPLSR) to select features and PLSR for classification to improve the performance of the PLSR classifier. The results of simulated and real fMRI data demonstrated that the PLSR classifier using PLSR/SPLSR to select features outperformed both the PLSR classifier using a general linear model (GLM) and the support vector machine (SVM) using PLSR/SPLSR/GLM in most cases. Moreover, PLSR using SPLSR to select features showed the best performance among all of the methods. Compared to GLM, PLSR is more sensitive in selecting the voxels that are specific to each task. The results suggest that the performance of the PLSR classifier can be largely improved when the PLSR classifier is combined with the feature selection methods of SPLSR and PLSR.

## Introduction

One of the key challenges in cognitive neuroscience is to map the human brain activities to different brain states. Multi-voxel pattern analysis (MVPA) using machine learning models has been widely applied to functional magnetic resonance imaging (fMRI) datasets to address this question [1]. The models that are trained on stimulus-evoked brain activity can be used to discriminate multiple cognitive processes [2–4].

Two critical steps that include feature selection and classification are involved in decoding brain states. Among the various MVPA methods, the partial least squares regression (PLSR) is a powerful method for multivariate data analysis that can be used in either feature/variable selection or classification. The variable selection methods based on PLSR can be categorized into three categories: filter, wrapper, and embedded methods [5]. In contrast to wrapper and embedded methods, filter methods that use the output of PLSR to identify a subset of important variables are fast and easy to compute. So far, PLSR filter methods have been applied to select features from fMRI data. Chou et al. (2014) used the magnitude of the PLS regression coefficient as an importance index to select features [6]. Swathi et al. (2015) proposed an effective feature (voxel) selection strategy that used PLSR to form an index for the informative voxels to prioritize the voxel selection based on the degree of association with the stimulus conditions [7]. Tu et al. (2016) applied PLSR to extract neural features that were correlated with the intensity of laser-evoked nociceptive pain from electroencephalography and fMRI data [8].

Except for feature/variable selection, PLSR can also be used for classification. After the PLS regression coefficients are estimated, some discriminate approaches on PLS-predicted response or scores are applied to fMRI-based decoding. Rodriguez (2010) applied the partial least squares (PLS) regression combined with a maximum output threshold to predict locations in the spatial navigation of humans with fMRI [9]. Moreover, PLSR using linear discriminant analysis (PLSLDA) was applied to decode emotional states from fMRI data [10] and to discriminate individuals with disease from those at high risk [11].

Although PLS has been widely applied to variable selection, it does not automatically select relevant variables. In recent years, sparse PLSR (SPLSR) methods that add sparse constraint to the PLSR have been proposed and applied to automatic variable selection [12] or classification [13]. However, SPLSR has seldom been applied to fMRI-based decoding. For fMRI data, the number of features is much larger than the number of samples. Because PLSR and SPLSR can manage a large number of features (p) and a small sample size (n), it is essential to investigate how PLSR and SPLSR should be applied effectively to fMRI-based decoding.

The previous studies applied PLSR/SPLSR to either feature selection or classification separately. It is unclear whether PLSR/SPLSR can be effectively used simultaneously for both feature selection and classification in fMRI-based decoding. In this study, we proposed a two-step PLSR framework that first used PLSR or SPLSR for feature selection and then used PLSR for classification. The method that uses PLSR for both feature selection and classification is named P_PLSR. The method that uses SPLSR for feature selection and PLSR for classification is named SP_PLSR. We investigated the robustness and feasibility of P_PLSR and SP_PLSR in brain-state decoding based on fMRI. Moreover, we compared P_PLSR and SP_PLSR using a general linear model (GLM) for feature selection and a support vector machine (SVM) for classification (G_SVM), PLSR for feature selection and SVM for classification (P_SVM), SPLSR for feature selection and SVM for classification (SP_SVM), and GLM for feature selection and PLSR for classification (G_PLSR). The results of both simulated and real fMRI data demonstrated that SP_PLSR and P_PLSR outperformed the other methods in most cases and that SP_PLSR showed the best decoding performance among the six methods.

## Theory

### PLSR

PLSR, which was first proposed by Wold et al. (1996), offers dimensionality reduction to solve ill-posed problems in econometrics and chemometrics [14]. In the context of fMRI data, suppose the matrix ***X***_***n***×***p***_ is fMRI data matrix with p voxels and n volumes (samples), and ***Y***_***n***×***q***_ is the class label with q classes from n samples. For multi-class (q¿2), ***Y*** needs to be transformed into dummy variables before PLSR computation [5].

$$Y_{ij}=\{\begin{array}{cc} 1, & \text{if}\;\text{the}\;i_{th}\;\text{sample}\;\text{belongs}\;\text{to}\;\text{the}\;j_{th}\;\text{category}\\ 0, & \text{otherwise} \end{array}$$

Each column of ***X*** and ***Y*** is centred. PLS regression is typically based on the basic latent component decomposition:

$$X=TQ_{X}^{T}+E_{X}$$ (1)

$$Y=TQ_{Y}^{T}+E_{Y}$$ (2)

where ***T***_***n***×***k***_ is a matrix with K latent components; ***Q***_***X***_ and ***Q***_***Y***_ are matrices of the loading coefficients; ***E***_***X***_ and ***E***_***Y***_ are matrices of random errors; and K is the number of latent components. The matrix T is defined as:

T=XW (3)

where ***W***_***p***×***k***_ contains K direction vectors. The prediction model can be expressed by Eq (4).

$$Y=X\beta +E_{Y}$$ (4)

where $\beta =WQ_{Y}^{T}$. Seeking the direction vectors *w*_(_ *k*) in ***W*** is equivalent to solving the following optimization problem:

$$maximize\;w_{\left(k\right)}^{T}Z^{T}Zw_{\left(k\right)}\;s.t.\;w_{\left(k\right)}^{T}\;w_{\left(k\right)}=1\;\text{and}\;w_{\left(k\right)}^{T}S_{X}w_{\left(k\right)}=0\;for\;all\;h<k$$ (5)

where ***Z*** = ***Y***^***T***^ ***X*** and ***S***_***X***_ = ***X***^***T***^ ***X***/*n*. ***S***_***X***_ is the sample covariance matrix of ***X***. The direction vector *w*_(*k*)_ can be obtained using the nonlinear iterative partial least squares (NIPALS) algorithm [14].

Let ***P***_***T***_ = ***TT***^+^ be the projection matrix on the space spanned by the columns of ***T***. ***T***^+^ is the Moore-Penrose inverse of ***T***. The NIPALS algorithm first finds the direction vector *r*_(*k*)_ with respect to the residual matrix $X_{\left(k\right)}=(I_{n}-P_{T_{\left(k-1\right)}})X$ at the *k*_*th*_ stage.

$$r_{\left(k\right)}=\text{arg}\;\text{max}\;r^{T}Z_{\left(k\right)}^{T}Z_{\left(k\right)}r\;s.t.\;r^{T}r=1$$ (6)

where $T_{\left(k-1\right)}=(X_{1}r_{1},\cdots X_{\left(k-1\right)}r_{\left(k-1)\right)},Z_{\left(k\right)}=Y_{\left(k\right)}^{T}X_{\left(k\right)},Y_{\left(k\right)}=(I_{n}-P_{T_{\left(k-1\right)}})Y$ and ***I***_***n***_ denotes the n × n identity matrix. The direction matrix ***W*** is computed by Eq (7).

$$W=R_{\left(K\right)}{\left(G_{\left(k\right)}^{T}R_{\left(K\right)}\right)}^{-1}$$ (7)

where $G_{\left(k\right)}=X^{T}T_{\left(K\right)}{\left(T_{\left(K\right)}^{T}T_{\left(K\right)}\right)}^{-1}$ and ***R***_(***K***)_ = (*r*_1_, ⋯, *r*_*K*−1_*r*_*K*_).

### SPLSR

For simplicity, the subscript (k) in the above section is omitted. In the NIPALS algorithm, the solution vector r can be regarded as the ordinary least squares estimator of the multivariate regression model in Eq (8) [15].

$$Z=Cr^{T}+e$$ (8)

where the response variable ***Z*** is the *q* × *p* matrix, the covariate *C* = *Y*^*T*^ *t* is a *q* × 1 vector, and the regression coefficient r is a *p* × 1 vector. Suppose the *j*_*th*_ columns of ***Z*** and ***e*** are *Z*_*j*_ and *e*_*j*_, respectively, and the *j*_*th*_ element of r is *r*_*j*_. Then, *r*_*j*_ can be estimated by the regression model in Eq (9).

$$Z_{j}=Cr^{j}+e$$ (9)

Based on the penalized least squares estimation of the regression model (9), SPLSR imposes sparseness on the PLS direction vectors by minimizing

$$Q_{\lambda }\left(r,Z\right)=\sum_{j=1}^{p}\left\{\frac{1}{2}{\left(Z_{j}-Cr_{j}\right)}^{T}\left(Z_{j}-Cr_{j}\right)+\lambda |r_{j}|\right\}$$ (10)

After the direction vector r is estimated by Eq (10), the direction matrix ***W*** can be calculated using Eq (7). The tuning parameters λ can be computed by the mean square error of cross-validation (MESCV). More mathematical details of SPLSR can be found in Lee’s study [16].

### PLSR and SPSR for feature selection

Both PLSR and SPLSR are based on the regression model in Eq (11).

$$Y_{n\times q}=X_{n\times p}B_{p\times q}$$ (11)

In the context of fMRI data, matrix ***X***_***n***×***p***_ is the observed fMRI data with p voxels and n volumes (samples). Suppose the fMRI data contains q tasks. ***Y***_***n***×***q***_ is the class label matrix. The element in the *j*_*th*_ column *y*_*j*_ of matrix ***Y*** is set to 1 when the corresponding volume of fMRI data ***X*** is induced by the *j*_*th*_ task. Otherwise, the element is set to zero. Matrix ***B***_***p***×***q***_ contains the regression coefficients that measure the intensity of the relationship between voxels and class label [17]. After ***B***_***p***×***q***_ is estimated by PLSR/SPLSR, the elements of each column of matrix ***B***_***p***×***q***_ are converted to Z scores. For the *j*_*th*_ task, the voxels whose z-score values in the *j*_*th*_ column of matrix ***B***_***p***×***q***_ are larger than 3.5 are selected as features relevant to the *j*_*th*_ task.

The features relevant to each task can be selected by applying PLSR/SPLSR to the whole brain fMRI data. The union of the features relevant to each task constitutes the final features.

### PLSR classifier

The selected features of the training data are inputted to PLSR to train the PLSR classifier based on the regression model in Eq (12).

$$Y_{n\times q}=X_{n\times k}B_{k\times q}$$ (12)

where k is the number of selected features. After the regression coefficient matrix, ***B***_***k***×***q***_, is estimated by PLSR, each test volume *D*_1×*k*_ is inputted to the classifier in Eq (13) using the same features as the training data to judge which task state it belongs to.

$$y_{1\times q}=D_{1\times k}B_{k\times q}$$ (13)

where *y*_1×*q*_ denotes weights of the q tasks. Finally, the test volume *D*_1×*k*_ is classified to the task whose weight in *y*_1×*q*_ is the largest in the q tasks(see Eq 14).

$$I\left(D_{1\times K}\right)=\underset{i}{\text{arg}\;\text{max}}\;y_{1\times i}.$$ (14)

Suppose the number of testing volumes is N and the number of testing volumes that are classified correctly is M. The classification accuracy can be calculated by the Eq 15.

$$R=\frac{M}{N}$$ (15)

## Materials and methods

We evaluated the robustness and feasibility of SP_PLSR and P_PLSR using both simulated and real fMRI data. Moreover, the voxel patterns that were selected by PLSR and GLM were compared, and the classification performances of SP_PLSR and P_PLSR were compared to G_PLSR, SP_SVM, P_SVM and G_SVM. The code of the PLSR algorithm was downloaded from http://www.utdallas.edu/~herve/, and the code of the SPLSR algorithm was downloaded from http://fafner.meb.ki.se/personal/yudpaw/?page_id=13. For PLSR and SPLSR, the number of the latent components is estimated by using the cross validation method. The SVM with a linear kernel was implemented using LIBSVM software (http://www.csie.ntu.edu.tw/\begingroup\let\relax\relax\endgroup[Pleaseinsert\PrerenderUnicode{}intopreamble]cjlin/libsvm/). The parameter c of SVM was set using the cross validation method provided by the LIBSVM software.

### Simulated experiment

#### Generation of simulated data

**Fig 1:**
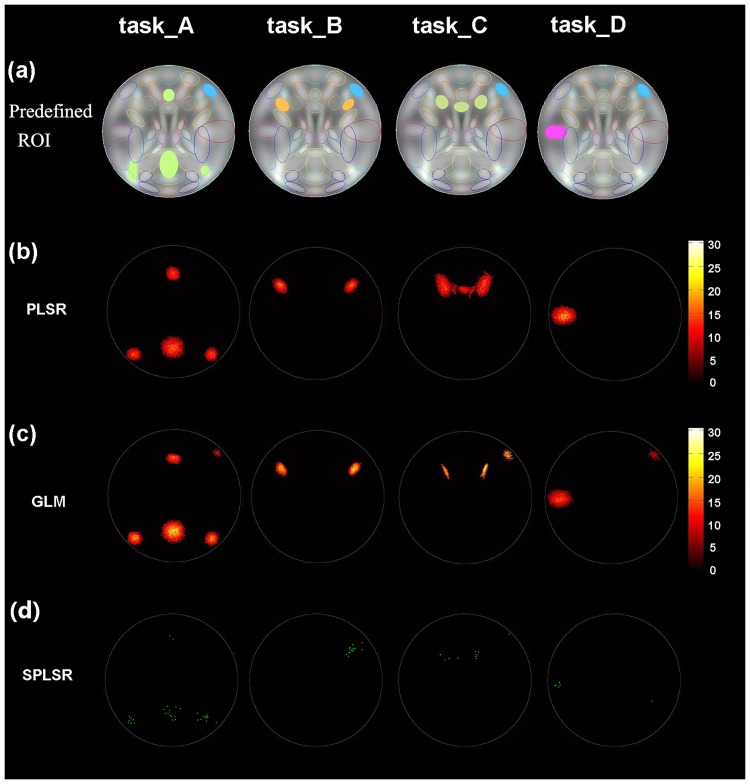
Pre-defined ROIs and voxel pattern. (a) Pre-defined ROIs of the simulated data. (b) The group voxel pattern relevant to each task of PLSR for the simulated dataset with CNR = 0.1. (c) The group voxel pattern relevant to each task of GLM for the simulated dataset with CNR = 0.1. (d) The voxel pattern relevant to each task of GLM for one subject’s simulated data with CNR = 0.1.

The four-task simulated datasets with different contrast-to-noise ratio (CNR) levels were generated using the simulation toolbox SimTB [18] (http://mialab.mrn.org/software/simtb/index.html). It was assumed that each simulated dataset consisted of two runs, and each run contained four tasks. For each run, sixteen 12-s task blocks alternated with seventeen 6-s rest blocks, with a repetition time (TR) of 2 s. Each task contained four blocks. The order of all the task blocks was randomized. The image of each time point had 270 × 270 pixels with a baseline intensity of 800. The regions of interest (ROI) that were activated by task A/B/C/D are shown in Fig 1a. The blue region in Fig 1a was supposed to be activated by the four tasks jointly. The simulated fMRI response of each task was derived from the convolution of the task paradigm and the hemodynamic response function (HRF) and was added to the corresponding ROIs in Fig 1. HRF was generated by using spm_hrf function with the default parameters in SPM8. Rician noise was added to each dataset relative to a specified CNR [18]. The CNR levels of the simulated datasets were set to 0.05, 0.1, 0.2 and 0.4. Fifteen simulated subjects were generated at each CNR level. In total, there were 60 simulated datasets (15 subjects × 4 CNR levels). Moreover, head motion was added to the simulated data by using the default parameters.

#### Voxel pattern analysis using PLSR, GLM and SPLSR

Simulated data with CNR = 0.1 were used for voxel pattern analysis. The time course of each voxel and the spatial pattern of each time point were normalized to a zero mean and a unit variance. GLM in the SPM8 software (http://www.fil.ion.ucl.ac.uk/spm/) was applied to each subject’s simulated data of the first run using the four tasks as the four regressors. Moreover, the first run of each subject was also inputted into PLSR/SPLSR to estimate the regression weights of each task. For each task, one-sample T test was applied to the corresponding weights of all the subjects to detect the voxels that were significantly relevant to each task at the group level after the regressor weights were estimated by GLM/PLSR. The statistical results were corrected by multiple comparisons using the cluster-size thresholding (p<0.001 at voxel level, cluster size>25). For SPLSR, the group voxel pattern cannot be obtained by using one-sample T test because the selected voxels were sparse and showed large variations across subjects. Each subject’s voxel patterns were obtained by selecting the voxels whose regression weights of SPLSR were larger than 3.5.

#### Classification

The simulated data of all CNR levels were used for classification. The time course of each voxel and the spatial pattern of each time point were normalized to a zero mean and a unit variance. For each subject’s simulated dataset, the first run was used as the training data, and the second run was used as the testing data. After GLM was applied to each subject’s training data, a T-test was performed on each voxel. The voxels whose T values of a specific task were significantly higher than the threshold (p<0.001 uncorrected) were selected as the features relevant to the task. Moreover, PLSR and SPLSR were applied to each subject’s training data to estimate the regression coefficient matrix ***B***. For each task, Z score transformation was applied to the corresponding column of the matrix ***B*** and the voxels with Z score higher than 3.5 were selected as the features relevant to the task. The union of the features relevant to each task were selected as the final features.

The features that were selected by GLM, PLSR and SPLSR were used to train two-class/three-class/four-class PLSR and SVM classifiers. After the SP_PLSR, P_PLSR, GLM_PLSR, GLM_SVM, SP_SVM, and P_SVM classifiers were trained, they were applied to the testing data to classify the task state of each volume. The testing samples used the same features as the training samples. For two/three/four-class PLSR classifiers, the test volume was classified to the task whose weight is the largest in the two/three/four tasks. For the SVM classifiers, six two-class SVM classifiers were trained first, and the trained two-class classifiers contributed to the final three/four-class classification via a simple voting mechanism. The accuracy of each subject was calculated using the ratio between the number of volumes that were correctly classified and the total number of volumes. For each classifier, the mean accuracy across the 15 participants at each noise level was obtained. To examine the difference of the classification accuracy between any two methods that used the same feature selection method or the same classifier, the nonparametric Wilcoxon signed rank tests for paired samples were performed in SPSS software. The significance level was set to P<0.05 for each statistical test.

### Real fMRI Experiment

The real fMRI experiment was conducted to further compare the performances of SP_PLSR, P_PLSR, GLM_PLSR, GLM_SVM, SP_SVM and P_SVM. The real fMRI data used in this study were the same as those used in our previous study [4]. The main points are repeated here.

#### Participants

Fifteen right-handed college students (age: 22.2±1.9 years) including eight females and seven males were recruited in this study. All participants gave written consent according to the guidelines set by the fMRI centre of Beijing Normal University. The experiment was approved by the Institutional Review Board (IRB) of the State Key Laboratory of Cognitive Neuroscience and Learning in Beijing Normal University.

#### Imaging parameters

Brain scans were performed using a 3.0-T Siemens whole-body MRI scanner. A single-shot T2*-weighted gradient-echo, EPI sequence was used for functional imaging acquisition with the following parameters: TR = 2000 ms, TE = 30 ms, flip angle = 90°, FOV = 190 × 200 mm, matrix = 64 × 64, and slice thickness = 1.33 mm. Thirty-two axial slices parallel to the line connecting the anterior and posterior commissures were obtained in an interleaved order to cover the entire cerebrum and part of the cerebellum.

#### Experimental design

The experiment included eight runs. There were four task blocks and five resting blocks in each run. In each 12-s task block, the participants were required to view 12 pictures from one of the four types (house, face, car and cat) and press the left button if any two sequential images were the same. During the 12-s control block, the subjects were required to view the fixation “+” on the screen.

#### Data pre-processing

Data pre-processing was performed in SPM8. The first three volumes of each run were discarded from each subject due to the instability of the initial scanning of each run. The functional images of each subject were realigned to correct the head motion, spatially normalized into the standard MNI template space, and resliced into 3 × 3 × 4 *mm*^3^ voxels.

#### Voxel pattern analysis using PLSR, GLM and SPLSR

For each pre-processed dataset, linear drift was removed using the spm_detrend function in SPM8. The time course of each voxel was normalized to a zero mean and a unit variance. For each dataset, we used the first four runs as training data and the last four runs as testing data. The group activity pattern of each task was detected by PLSR and GLM using the same method as that for the simulated data. For SPLSR, each subject’s voxel patterns were obtained by selecting the voxels whose regression weights of SPLSR were larger than 3.5.

#### Classification

For the two-class/three-class/four-class classification, the training data and testing data contained the samples of two/three/four tasks. Feature selection and classifier training were performed in the same way as that for the simulated datasets. For each classifier, the mean accuracy across the 15 participants was obtained. To examine the difference in classification accuracy between any two methods that used the same feature selection or classifier, nonparametric Wilcoxon signed rank tests for paired samples were performed. The significance level was set as P<0.05 for each test.

## Results

### Simulated experiment

#### Voxel pattern analysis using PLSR, GLM and SPLSR

The group voxel pattern of the simulated data with CNR = 0.1 using PLSR and GLM are displayed in Fig 1(b) and 1(c). Compared to the pre-defined ROIs in Fig 1a, PLSR detected regions that were only activated by each task and did not detect regions that were jointly activated by the four tasks. In contrast, GLM detected regions that were specific to each task and regions that were common to the four tasks. Moreover, it can be seen that regions detected by GLM showed larger overlap than that of regions detected by PLSR. For SPLSR, the voxels relevant to each task of one subject are shown in Fig 1d. It can be seen that the voxels selected by SPLSR were sparse and located in the pre-defined ROIs of each task.

#### Classification

**Fig 2:**
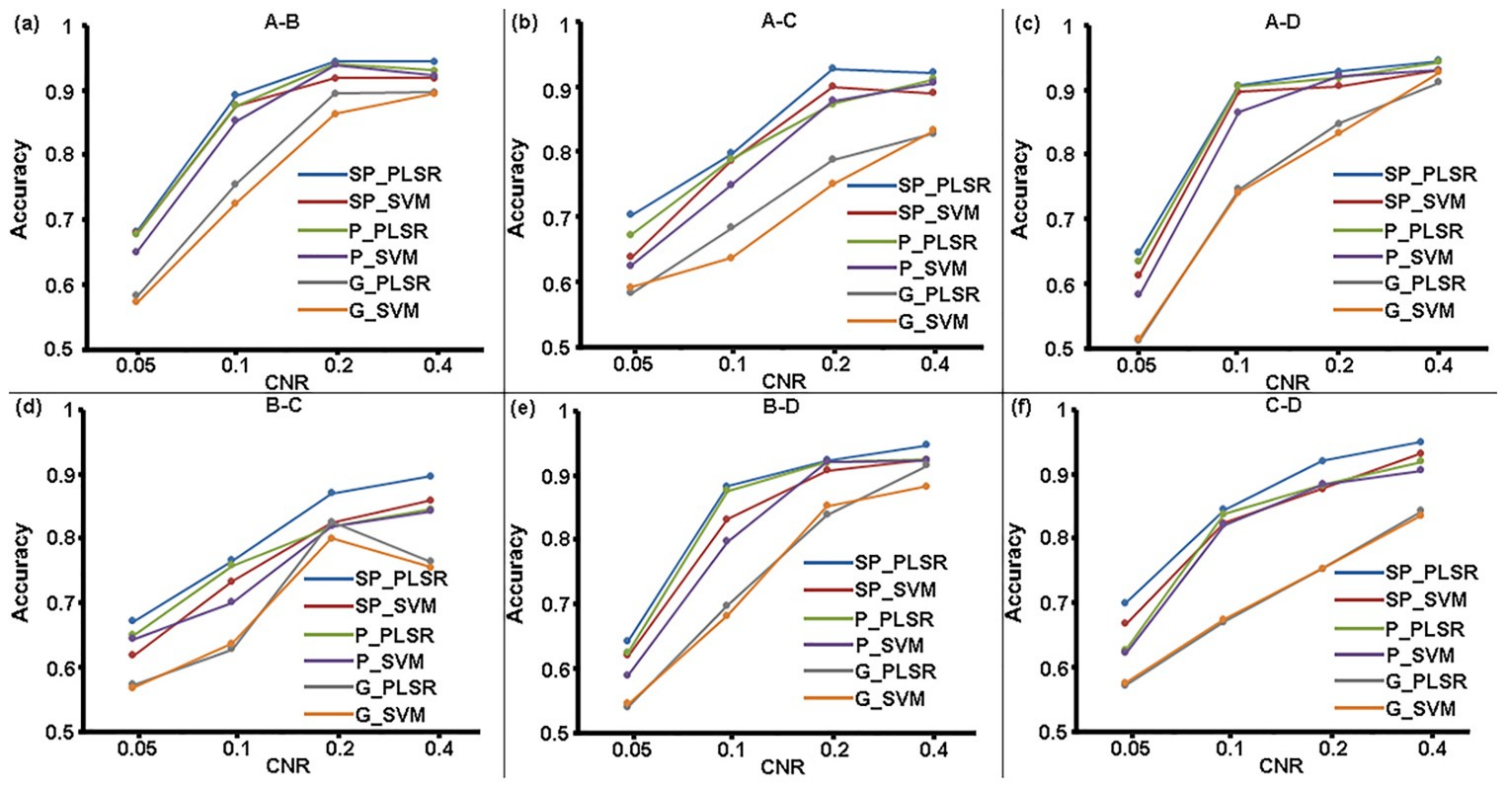
The accuracies of the two-class classifications at the four CNR levels for the six classifiers.

**Fig 3:**
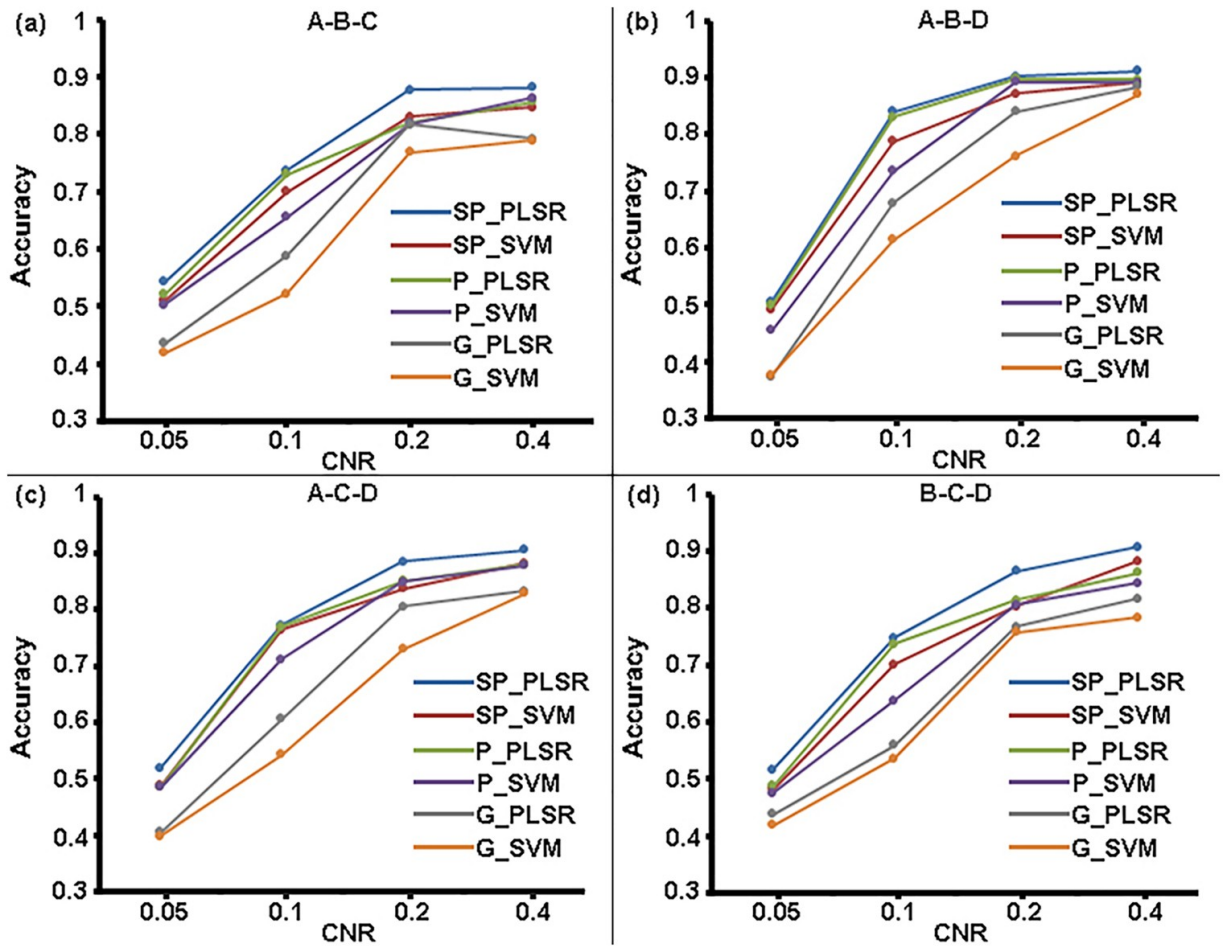
The accuracies of the three-class classifications at the four CNR levels for the six classifiers.

**Fig 4:**
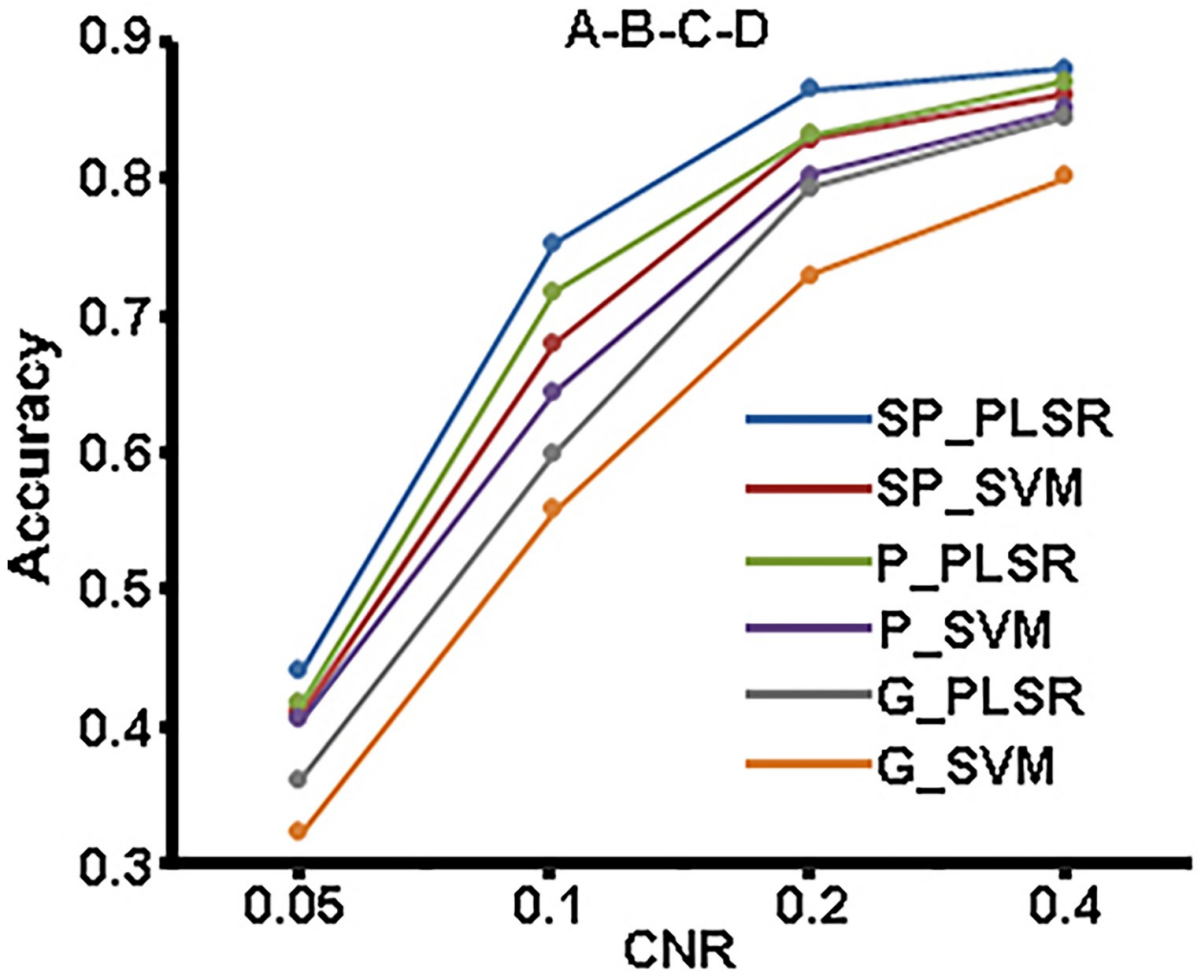
The accuracies of the four-class classifications at the four CNR levels for the six classifiers.

The mean classification accuracies of the two-class, three-class and four-class classifications at various CNR levels for SP_PLSR, SP_SVM, P_PLSR, P_SVM, G_PLSR, and G_SVM are shown in Figs 2–4. It can be seen that the mean classification accuracies of all six methods increased with increasing CNR. Among the six methods, SP_PLSR showed the highest accuracies while G_SVM showed the lowest accuracies for all of the classifications. Moreover, the accuracies of P_PLSR were higher than those of SP_SVM, P_SVM, G_PLSR and G_SVM in most cases. G_SVM and G_PLSR that used GLM to select features produced lower accuracies than those of the other methods that used SPLSR or PLSR to select features, in all cases. SP_PLSR/P_PLSR/GLM_PLSR using the PLSR classifier showed higher accuracies than SP_SVM/P_SVM/GLM_SVM using the SVM classifier when PLSR and SVM used the same feature selection method.

### Real fMRI Experiment

#### (1) Voxel pattern analysis using PLSR, GLM and SPLSR

**Fig 5:**
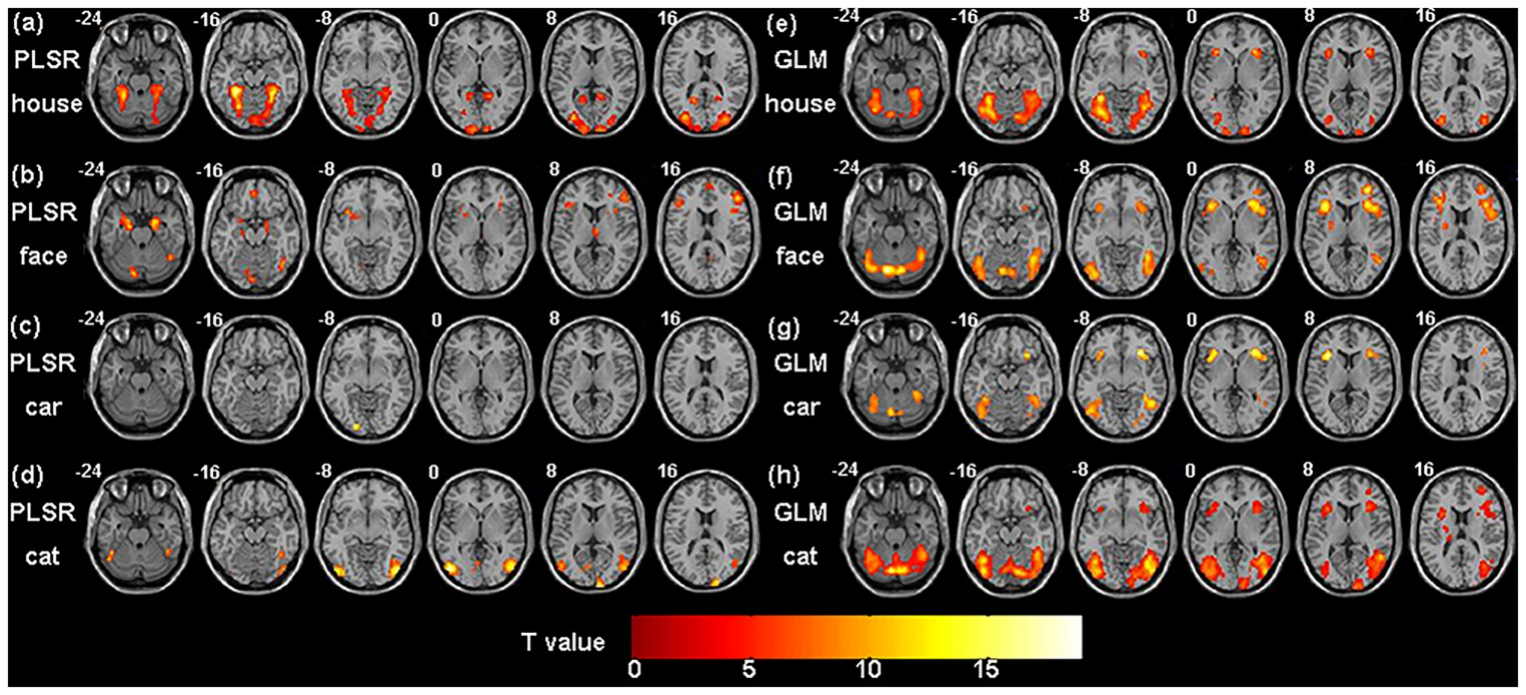
The group voxel pattern relevant to each task for the real fMRI data. (a-d) PLSR (e-h) GLM.

The group voxel pattern of each task for PLSR and GLM are displayed in Fig 5. The local maxima coordinates of the activation of PLSR are reported in Table 1 and those of GLM are reported in Table 2.

**Table 1:** Activity foci (MNI coordinates) of each task detected by PLSR.

*Method*	*Task*	*Regions*	*L/R*	*BA*	*Peak MNI Coordinates*			
					*X*	*Y*	*Z*	*t*_*max*_
PLSR	house	Fusiform gyrus	L	37	-27	-43	-18	19.00
		Parahippocampa gyrus	L	36	-27	-37	-14	12.41
		Middle Occipital gyrus	L	19	-30	-85	22	11.55
		Lingual	L	30	-15	-52	2	8.46
		Fusiform gyrus	R	37	30	-46	-14	14.32
		Parahippocampa gyrus	R	19	36	-40	-10	11.13
		Middle Occipital gyrus	R	19	33	-88	22	10.84
		Lingual	R	30	18	-49	2	8.57
	face	Amygdala	L	34	-21	-1	-18	8.71
		Hippocampus	L	34	-18	-10	-18	9.74
		Insula	L	47	-33	17	-6	7.32
		Fusiform gyrus	R	37	45	-61	-14	9.34
		ParaHippocampal gyrus	R	19	18	-7	-22	9.16
		Inferior frontal gyrus	R	46	51	38	14	7.06
		Inferior occipital gyrus	R	19	42	-70	-18	8.86
	car	Inferior occipital gyrus	L	17	-18	-91	-6	10.45
	cat	Fusiform gyrus	L	37	-45	-58	-18	7.99
		Inferior occipital gyrus	L	18	-42	-79	-6	7.21
		Middle occipital gyrus	L	19	-48	-79	2	10.35
		Middle temporal gyrus	L	37	-51	-67	6	7.10
		Inferior occipital gyrus	R	19	39	-88	-10	8.62
		Inferior Temporal gyrus	R	37	51	-70	-6	8.35
		Middle Temporal gyrus	R	37	57	-64	6	9.37

**Table 2:** Activity foci (MNI coordinates) of each task detected by GLM.

*Method*	*Task*	*Regions*	*L/R*	*BA*	*Peak MNI Coordinates*			
					*X*	*Y*	*Z*	*t*_*max*_
PLSR	house	Fusiform gyrus	L	37	-30	-70	-10	16.15
		Parahippocampa gyrus	L	19	-27	-43	-10	7.18
		Inferior frontal gyrus	L	47	-33	26	6	8.11
		Middle occipital gyrus	L	19	-45	-70	-10	8.44
		Fusiform gyrus	R	37	36	-49	-14	7.22
		Parahippocampa gyrus	R	19	30	-43	-10	7.29
		Inferior frontal gyrus	R	47	33	26	-2	7.45
		Middle occipital gyrus	R	19	39	-85	10	7.21
	face	Fusiform gyrus	L	37	-39	-52	-22	7.32
		Inferior occipital gyrus	L	19	-39	-79	-6	8.06
		Fusiform gyrus	R	37	45	-55	-18	7.55
		Inferior occipital gyrus	R	19	45	-76	-10	8.34
		Inferior temporal gyrus	R	19	48	-49	-10	7.73
		Inferior frontal gyrus	R	47	48	17	2	9.79
		Middle frontal gyrus	R	10	33	47	10	12.14
	car	Fusiform gyrus	L	37	-36	-52	-10	7.33
		Inferior temporal gyrus	L	19	-45	-67	-10	8.45
		Inferior frontal gyrus	L	47	-30	26	6	7.98
		Inferior temporal gyrus	R	19	48	-52	-10	7.61
		Inferior frontal gyrus	R	47	33	26	6	7.95
	cat	Fusiform gyrus	L	37	-45	-58	-18	9.2
		Inferior occipital gyrus	L	19	-42	-85	-1	7.5
		Inferior temporal gyrus	L	19	-42	-61	-10	12.99
		Insula	L	13	-30	20	14	8.16
		Fusiform gyrus	R	37	45	-58	-18	9.44
		Inferior occipital gyrus	R	19	39	-82	-14	7.93
		Inferior temporal gyrus	R	19	42	-61	-6	11.39
		Insula	R	13	33	20	10	7.07
		Middle occipital gyrus	R	19	42	-79	2	7.67
		Middle frontal gyrus	R	10	36	47	14	9.18

For the house task, PLSR mainly detected activation in the bilateral fusiform gyrus, bilateral para-hippocampal gyrus, bilateral middle occipital gyrus and bilateral lingual, whereas GLM mainly detected activation in the bilateral fusiform gyrus, bilateral para-hippocampal gyrus, bilateral inferior frontal gyrus and bilateral middle occipital gyrus.

For the face task, PLSR mainly detected activation in the left amygdala, left hippocampus, left insula, right fusiform gyrus, right para-hippocampal gyrus, right inferior frontal gyrus and right inferior occipital gyrus, whereas GLM mainly detected activation in the bilateral fusiform gyrus, bilateral inferior occipital gyrus, right inferior temporal gyrus, right inferior frontal gyrus and right middle frontal gyrus.

For the car task, PLSR mainly detected activation in the left inferior occipital gyrus, whereas GLM mainly detected activation in the left fusiform gyrus, bilateral inferior temporal gyrus and bilateral inferior frontal gyrus.

For the cat task, PLSR mainly detected activation in the left fusiform gyrus, bilateral inferior occipital gyrus, bilateral middle temporal gyrus, left middle occipital gyrus and right inferior temporal gyrus, whereas GLM mainly detected activation in the bilateral fusiform gyrus, bilateral inferior occipital gyrus, bilateral inferior temporal gyrus, bilateral insula, right middle occipital gyrus and right middle frontal gyrus.

It can be seen that the activated regions of each task showed little overlap for PLSR and showed large overlap for GLM. Thus, PLSR was more sensitive than GLM in detecting the brain regions that were specific to each task.

**Fig 6:**
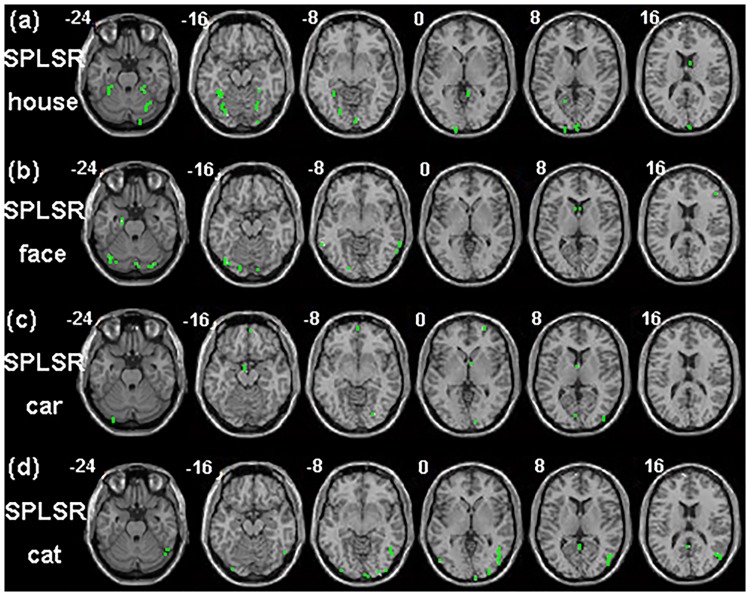
The voxel pattern relevant to each task of SPLSR for one subject’s real fMRI data.

For SPLSR, the voxels relevant to each task of one subject are shown in Fig 6. The voxel pattern of each task was sparse distribution and the selected voxels located in the regions that were detected by PLSR and GLM.

#### Classification

**Fig 7:**
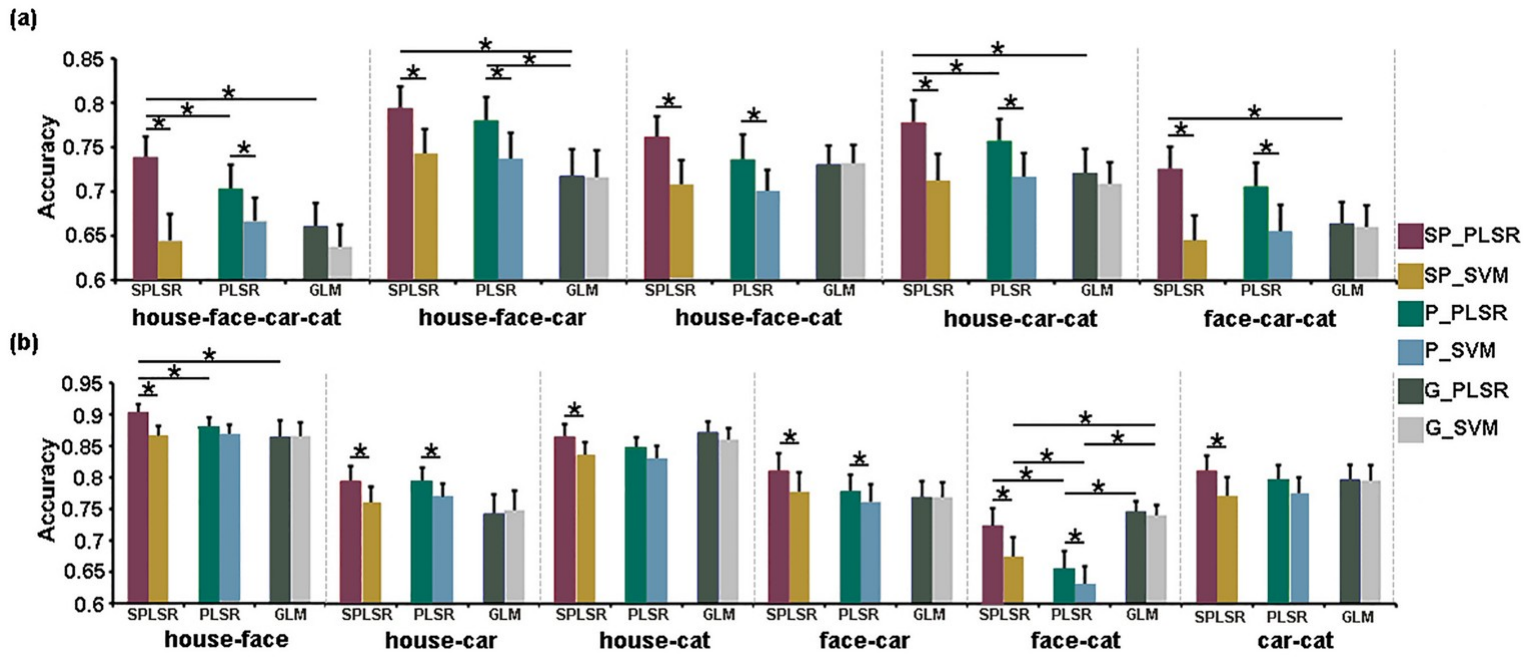
The accuracies of the two-class, three-class and four-class classifications for the real fMRI data.

The mean classification accuracies of the four-class, three-class and two-class classifications using SP_PLSR, SP_SVM, P_PLSR, P_SVM, G_PLSR, G_SVM are shown in Fig 7. Among the six methods, SP_PLSR showed the highest classification accuracy in most cases.

For the four-task classification, SP_PLSR exhibited significantly higher classification accuracy than SP_SVM, P_PLSR and G_PLSR. The accuracy of P_PLSR was significantly higher than that of P_SVM.

For the three-task classification, SP_PLSR exhibited significantly higher classification accuracy than SP_SVM in all cases and G_PLSR in most cases. Moreover, the accuracy of SP_PLSR was significantly higher than that of P_PLSR for house vs. car vs. cat. The accuracy of P_PLSR was significantly higher than that of P_SVM in all cases and that of G_PLSR in the case of house vs. face vs. car.

For the two-task classification, SP_PLSR exhibited significantly higher classification accuracy than SP_SVM in all cases. Moreover, the accuracy of SP_PLSR was significantly higher than that of G_PLSR and P_PLSR in the case of house vs. face.

## Discussion

In this study, we proposed a two-step PLSR framework and investigated the performance of SP_PLSR and P_PLSR in fMRI-based decoding. Moreover, SP_PLSR and P_PLSR were compared with GLM_PLSR, GLM_SVM, SP_SVM and P_SVM using both simulated and real fMRI data. The results indicated that SP_PLSR produced the best decoding performance among all the methods and that P_PLSR outperformed GLM_PLSR, GLM_SVM, SP_SVM and P_SVM in most cases. Compared to GLM, the feature selection method of PLSR was more sensitive in detecting voxels that were specific to a task.

Both spatial maps of the features that were selected by PLSR and GLM showed clustered distribution while the voxels that were selected by SPLSR showed sparse distribution. In the simulated experiment, PLSR detected voxels that were relevant to each task only and could not detect voxels that were relevant to all the tasks (see Fig 3). In contrast, GLM detected voxels that were either specific to each task or common to all the tasks. In the real fMRI experiment, PLSR detected the bilateral para-hippocampal place area (PPA) in the house task and right fusiform face area (FFA) in the face task. It has been demonstrated that PPA plays an important role in the encoding and recognition of environmental scenes [19] and that FFA is important to face perception [20]. Thus, PPA is specific to house processing, and FFA is specific to face processing. The regions relevant to each task showed slight overlaps for PLSR and large overlaps for GLM. Because the voxels that are common to all tasks may provide less discriminative information voxels than those that are specific to each task, P_PLSR showed higher classification accuracy than G_PLSR in most cases. In contrast to PLSR and GLM, SPLSR selected fewer voxels and selected the voxels relevant to each task only. (see Figs 1d and 6).

The classifier PLSR was more accurate than SVM in both simulated and real fMRI experiments when using SPLSR/PLSR as the feature selection method. Although the accuracies of G_PLSR were better than those of G_SVM in the simulated data, the two methods did not show significant differences in the real fMRI data. Thus, the PLSR classifier showed better decoding performance than SVM when SPLSR and PLSR were used as the feature selection methods. The advantages of the PLSR classifier over the SVM was weakened when using GLM as the feature selection method. The results may indicate that the features selected by SPLSR and PLSR were more suitable to the PLSR classifier than those selected by SVM.

In the simulated experiment, SP_PLSR produced higher accuracy than the other methods at all SNR levels. The classification accuracies of P_PLSR were higher than those of GLM_PLSR, GLM_SVM, SP_SVM and P_SVM at the low SNR levels. In the real experiment, SP_PLSR and P_PLSR outperformed the other methods, mainly in the multi-class classification. The advantages of SP_PLSR over P_PLSR were weakened for the real data. The results indicated that the proposed two-step PLSR methods, including both SP_PLSR and P_PLSR, have better robustness to noises than do GLM_PLSR, GLM_SVM, SP_SVM and P_SVM. PLSR was seldomly used as a classifier to directly decode the fMRI data. Rodriguez (2010) applied PLSR to decode the goal locations in the spatial navigation in humans with fMRI and obtained higher decoding accuracies than the naïve classifier did [9]. This study further indicated that the performance of the PLSR classifier can be improved when the feature selection method PLSR/SPLSR is combined with the PLSR classifier. It has been demonstrated that PLSR is very resistant to overfitting [21]. When PLSR was used in both feature selection and classification, the advantages of PLSR were further strengthened and led to an improvement in the decoding performance of P_PLSR. In contrast to PLSR, SPLSR is able to select more discriminative features because the sparse regularization can remove irrelevant features. Therefore, SP_PLSR showed more stable and better performance than P_PLSR did.

It should be noted that deep learning methods have attracted more and more attentions in brain imaging classification [22, 23]. Although deep learning methods achieved promising results in classification of cognitive disorders using brain imaging techniques, its decoding performance depends on the number of training samples. In this study, it is impossible to collect large training samples of fMRI data from one subject. Thus, the deep learning methods do not fit for decoding brain state in this study.

Moreover, it has been demonstrated that head motion may have both noisy and neuronal effect on fMRI measures [24, 25]. Although the motion was corrected in the preprocessing steps, the residual of head motion cannot be totally removed from fMRI data. Thus, head motion may be one of the confounding factors in the voxel pattern classification. It is necessary to further investigate how the head motion will affect the brain-state decoding of fMRI in our future studies.

## Conclusion

This study proposed two-step PLSR methods (SP_PLSR and P_PLSR) and applied them to fMRI-based decoding. Simulated and real fMRI data demonstrated that both SP_PLSR and P_PLSR showed better robustness to noises and were more accurate than SP_SVM, P_SVM, G_SVM and G_PLSR in most cases. Moreover, SP_PLSR performed better than P_PLSR in most cases. The results suggest that the performance of the PLSR classifier can be largely improved when it is combined with the feature selection methods of SPLSR and PLSR.
